# Duplex real-time PCR for sexing *Schistosoma japonicum* cercariae based on W chromosome-specific genes and its applications

**DOI:** 10.1371/journal.pntd.0008609

**Published:** 2020-08-21

**Authors:** Shuai Liu, Xianyu Piao, Nan Hou, Pengfei Cai, Yu Ma, Qijun Chen

**Affiliations:** 1 NHC Key Laboratory of Systems Biology of Pathogens, Institute of Pathogen Biology, Chinese Academy of Medical Sciences and Peking Union Medical College, Beijing, P.R. China; 2 Molecular Parasitology Laboratory, QIMR Berghofer Medical Research Institute, Brisbane, Australia; 3 Key Laboratory of Livestock Infectious Diseases in Northeast China, Ministry of Education, Key Laboratory of Zoonosis, College of Animal Science and Veterinary Medicine, Shenyang Agriculture University, Shenyang, P.R. China; Stanford University Hopkins Marine Station, UNITED STATES

## Abstract

As a unique feature among otherwise hermaphroditic trematodes, *Schistosoma* species are gonochoric parasites whose sex is genetically determined (ZZ for males and ZW for females). However, schistosome larvae are morphologically identical, and sex can only be discriminated by molecular methods. Here, we integrated published *Schistosoma*. *japonicum* transcriptome and genome data to identify W chromosome-specific genes as sex biomarkers. Three W chromosome-specific genes of *S*. *japonicum* were identified as sex biomarkers from a panel of 12 genes expressed only in females. An efficient duplex real-time PCR (qPCR) method for sexing cercariae was developed which could identify the sex of cercariae within 2 h without DNA extraction. Moreover, this method can be used to identify not only single-sex but also mixed-sex schistosome-infected snails. We observed a nearly equal proportion of single-male, single-female, and mixed-sex schistosome infections in artificially infected snails. Sex-known schistosome-infected snail models can be efficiently constructed with the aid of duplex qPCR. A field study revealed that single-sex schistosome infections were predominant among naturally infected snails. Finally, a schistosomiasis mouse model based on sex-known cercariae infection was shown to be more reliable than a model based on sex-unknown cercariae infection. The developed duplex qPCR method for sexing *S*. *japonicum* cercariae can be widely used for schistosomiasis modeling, genetic experiments, and field-based molecular epidemiological studies.

## Author summary

*Schistosoma japonicum* is a major causative agent of human schistosomiasis. Unlike other parasitic worms, *S*. *japonicum* females are determined by the heterogametic sex chromosome (ZW) and males by the homogametic sex chromosome (ZZ). The life cycle of *S*. *japonicum* includes the egg, miracidium, mother sporocyst, daughter sporocyst, cercaria, schistosomulum, and adult stages. The sex of adult male and female worms can be morphologically distinguished, whereas the sex of larvae, such as cercariae, can only be discriminated by molecular methods. In this study, we established an efficient duplex real-time PCR method for sexing *S*. *japonicum* cercariae based on newly identified W chromosome-specific genes. The established duplex real-time PCR method will facilitate construction of sex-controlled schistosome-infected intermediate host or definitive host models for schistosome-host interplays and schistosomiasis studies. This method is also a powerful tool for investigating the epidemiology of single-sex and mixed-sex schistosome-infected snails in the field.

## Introduction

Schistosomiasis affects approximately 200 million people worldwide and, according to conservative estimates, translates into a global burden of more than one million years lived with disability [1]. *Schistosoma japonicum*, *Schistosoma mansoni*, and *Schistosoma haematobium* are the major causative agents of human schistosomiasis [2]. Besides the medical importance of *Schistosoma* spp. in human health, these parasites are very intriguing as biological, genetic, and evolutionary models [3, 4]. The life cycle of schistosomes comprises seven morphologically discrete stages: egg, miracidium, mother sporocyst, daughter sporocyst, cercaria, schistosomulum, and adult. Schistosomes need two obligatory hosts: a molluscan intermediate host, in which the parasites multiply asexually, and a mammalian definitive host, in which the adult parasites reproduce sexually [5].

As a unique feature among more common hermaphroditic trematodes, schistosomes are gonochoric parasites characterized by morphological, ecological, behavioral, and molecular differences between males and females throughout their life cycle [5]. The sex of schistosomes is genetically determined and is described as ZZ for males and ZW for females. Whereas adult male and female worms can be distinguished morphologically [3], the sex of larvae can be identified only based on W chromosome-specific DNA sequences. Several PCR approaches have been developed to determine the sex of *S*. *mansoni* or *S*. *haematobium* larvae [6–11], but only a few molecular methods for sexing *S*. *japonicum* larvae have been developed so far [12]. The published draft genomes of the three major *Schistosoma* species contain a wealth of information for studies on molecular parasitology, host-parasite interplays, as well as anti-parasite drug and vaccine targets [13–15]. Recently, the draft genomes of *S*. *japonicum* and *S*. *haematobium* were further improved by third-generation sequencing technology [16, 17]. Unfortunately, the available genome of *S*. *japonicum* remains at the scaffold level, making it difficult to identify W chromosome-specific genes or DNA sequences based on its fragmentary genome sequences. In our previous study, we constructed a next-generation microarray to investigate the expression of sex-biased genes in *S*. *japonicum*, and identified hundreds of genes expressed only in adult females [18]. We hypothesized that some of these genes are located exclusively on the W chromosome.

Based on the above data as well as published transcriptome and genome databases of *S*. *japonicum*, we aimed to identify W chromosome-specific genes that could be used as sex biomarkers. An efficient duplex real-time PCR (qPCR) for sexing *S*. *japonicum* cercariae was established using these biomarkers. The duplex qPCR was used to construct a controllable schistosome-infected snail model and schistosomiasis mouse model, and investigate the epidemiology of single- or mixed-sex schistosome infections of snails in an endemic lake region in China.

## Materials and methods

### Ethics statement

All procedures performed on animals in this study were conducted following animal husbandry guidelines set by the Chinese Academy of Medical Sciences and with permission from the Experimental Animal Committee (Institute of Pathogen Biology, CAMS) with Ethical Clearance Number IPB-2011-6.

### Snails and parasites

*Oncomelania hupensis* snails artificially infected with *S*. *japonicum* were provided by Anhui Institute of Parasitic Diseases and Jiangsu Institute of Parasitic Diseases, China. *Oncomelania*. *hupensis* snails naturally infected with *S*. *japonicum* were provided by Hunan Institute of Schistosomiasis Control and Jiangxi Institute of Parasitic Diseases, China. Cercariae were shed by exposing the snails to light for 2 h. BALB/c mice (6 weeks old, female) were percutaneously infected with ∼400 (for isolation of hepatic schistosomula) or ∼40 cercariae (for isolation of adult worms). Hepatic schistosomula were isolated from mice at 2 weeks post-infection, while adult worms were obtained at 6 weeks post-infection by hepatic-portal perfusion [19]. Parasites used for genomic DNA isolation were soaked in absolute alcohol, and stored at -80°C. Parasites intended for total RNA isolation were soaked in RNAlater solution (Ambion, CA, USA) overnight at 4°C; the solution was discarded, and the samples were stored at -80°C.

### cDNA and genomic DNA

Total RNA was extracted from ~30 adult male or adult female worms using an RNeasy Mini Kit (QIAGEN, Hilden, Germany), and potentially contaminating genomic DNA was removed using a TURBO DNA-free Kit (Ambion, CA, USA). Next, 1 μg of male or female total RNA was reverse-transcribed into the first strand cDNA using a SuperScript III Reverse Transcriptase Kit (Invitrogen, CA, USA) with oligo (dT) 15 primer. Genomic DNA was isolated from ~30 adult male or adult female worms with a QIAamp DNA Mini Kit (QIAGEN). All experiments were performed according to the manufacturer’s instructions.

### Selection of candidate genes for W chromosome-specific gene screening

Candidate genes were selected according to the following criteria: genes were selected from the top 100 adult female-only expressed genes based on our published microarray data [18]; the genes’ mRNA sequence should be well mapped to the published *S*. *japonicum* genome [13] with coverage ≥85% and identity ≥99%; genes should be annotated with biological function.

### End-point PCR for identifying W chromosome-specific genes

For all genes, primer pairs were designed using Primer Premier 5.0 software (PREMIER Biosoft, CA, USA) (Table 1). PCR was carried out in a 25 μL total volume containing 12.5 μL Q5 High-Fidelity DNA Polymerase mix (NEB, Ipswich, MA, USA), 1 μL cDNA or DNA (10 ng), 1 μL forward and reverse primer pair (at 5 μM each), and 10.5 μL sterile water. The 26S proteasome non-ATPase regulatory subunit 4 gene (*PSMD4*), which has been validated as a reliable reference gene in *S*. *japonicum* [20], was employed as a PCR internal control (forward primer sequence 5′-CCTCACCAACAATTTCCACATCT-3′ and reverse primer sequence 5′-GATCACTTATAGCCTTGCGAACAT-3′). The PCR cycling conditions were as follows: 98°C for 1 min, followed by 30 cycles of denaturation at 98°C for 10 s, annealing at 60°C for 10 s, and extension at 72°C for 5 s. PCR products were examined by 2.5% agarose gel electrophoresis. W chromosome-specific genes were identified based on the fact that PCR products could only be amplified from both cDNA and genomic DNA of females. As a negative control, PCR was performed using DNase-treated RNA from male and female worms as template, and no PCR products of *PSMD4* and selected candidate genes (Table 1) were detected, confirming that genomic DNA was completely removed from the extracted RNA samples (S1 Fig).

**Table 1:** Summary information of 12 candidate genes for sex biomarker screening.

Candidate gene	Annotation	GenBank	Genome scaffold	Primer sequence (5'-3')	Amplicon length (bp)	
				Forward / Reverse	cDNA	gDNA
*SjF1*	Histidine-rich glycoprotein precursor	AY812810.1	FN331446.1	GATGAATATGAACATCCACGTCA	129	129
				ATGATATCGATGTGTTGGTGATG		
*SjF2*	Asparagine-rich antigen Pfa35-2	FN314868.1	FN335096.1	CTGATGAGCCTCACACAGACGA	150	150
				CATAATAGTAGTCACCTTCCGAACC		
*SjF3*	Female-specific protein 800	AY815418.1	FN339443.1	ATGGTTCATCCAAAGGTTATCG	163	163
				ACCTCACTGTTGTTAGGCGAAT		
*SjF4*	Splicing factor U2AF 65 kDa subunit	FN317243.1	FN342316.1	AGATGGCTCAACTGGTCTATCA	190	190
				ACCAGACATTTGCAGCAGACAC		
*SjF5*	CLECT Superfamily member	AY813874.1	FN331200.1	CAAGACAATTTTGGATAGGTGG	201	201
				CACCCCATTTACCACTAGCAT		
*SjF6*	UV excision repair protein RAD23	AY811322.1	FN341289.1	GAATTCGTTGCAAGCGCTCAA	211	260
				TGGGACTCTTTTCACTTGGCA		
*SjF7*	Extracellular superoxide dismutase [Cu-Zn]	FN316055.1	FN331319.1	GGAAAGCCTAGACATGCTGGT	216	216
				AGCACAAGCTAATCTCGGACC		
*SjF8*	Globin-3	FN319742.1	FN332322.1	GTCACTCAATCCCAGGTAGATC	224	260
				GGAACAAAAGCAGCAACGAAAG		
*SjF9*	Histone H3.3	FN319455.1	FN344783.1	GCGTCCAAATTTCGTGAGAT	226	226
				TTTGAAACGGCAACTTTCGG		
*SjF10*	Beta/gamma crystallin	FN313788.1	FN354665.1	CTCATTTCAGAGGACCCTACCTG	265	265
				GTTAAACTACCCGATGGATTGACT		
*SjF11*	TES domain-containing protein	FN315504.1	FN335014.1	CAAATCAAGGTTCAGACTACGC	277	277
				TATGGATGTCCGACTGGTGAG		
*SjF12*	Tyrosinase	AY815264.1	FN332875.1	TGTTTCCCTACATGGCATCGTT	290	290
				GTTCCTCTATTACCACCTCTTTG		

Previous studies have reported a high degree of genetic diversity among different geographical strains or lineages of *S*. *japonicum* [21–24]. To confirm that the primer pairs for W chromosome-specific genes worked consistently across different parasite populations, a set of duplex PCRs was carried out using genomic DNA of individual adult male and female worms (from four different endemic regions of China, including two strains maintained in the laboratory (Anhui and Jiangsu provinces) and two strains collected from the field (Hunan and Jiangxi provinces). All PCR amplifications were performed in biological triplicate for each sample. The PCR reactions and cycling conditions were as described above. PCR products were examined by 2.5% agarose gel electrophoresis.

### Gene expression analysis

Gene expression patterns of candidate genes (Table 1) at different developmental stages (egg, cercaria, schistosomulum, and adult) were analyzed by qPCR as previously described [18]. Reactions were carried out in technical triplicates on a 7300 Real-Time PCR system (Applied Biosystems, CA, USA) using Brilliant II SYBR Green QPCR Master Mix (Agilent Technologies, CA, USA) according to the manufacturer’s instructions. qPCR cycling conditions were as follows: 95°C for 10 min, followed by 40 cycles of 30 s denaturation at 95°C and 1 min annealing and extension at 60°C. A dissociation step (95°C for 15 s, 60°C for 1 min, 95°C for 15 s, and 60°C for 15 s) was performed to confirm the amplification specificity for each gene. Melting curves for the tested genes are shown in S2 Fig. *PSMD4* was employed as a control gene [20]. The relative expression level of each gene was analyzed by comparative 2^-ΔΔCt^ method in SDS 1.4 software (Applied Biosystems).

### Single-sex schistosome-infected snail models

Miracidia were hatched from eggs isolated from livers of 42-day post-infected mice. Each snail was immersed in water containing one miracidium per well in a 96-well plate at 26°C for 1 h. The snails were incubated at 26°C for 3 months, and cercariae were released by one snail per well in a 24-well plate under light stimulation. Mice were infected with freshly released cercariae as following: one mouse was percutaneously infected with ~50 cercariae released from a single snail. The sex of cercariae released from each snail was confirmed by morphologically distinguishable adult worms isolated from the mice infected with cercariae from a single snail [25].

### Duplex qPCR for sexing cercariae without DNA isolation

Duplex qPCR was performed in 8-strip PCR tubes using the 7300 Real-Time PCR system. Single male or female cercariae were freshly released from single-sex schistosome-infected snails in a 24-well plate. With the aid of an anatomical microscope, three randomly selected cercariae were pipetted into PCR tubes (one cercaria per tube) containing 5 μL of 10-fold diluted proteinase K (QIAGEN). Then, the cercariae were lysed at 60°C for 5 min by proteinase K to release genomic DNA, after which proteinase K was inactivated by incubation at 98°C for 10 min. Finally, 20 μL of PCR reaction mixture was added to each tube. The mixture contained 12.5 μL of 2× TB Green Premix Ex Taq (Takara Bio, Dalian, China), 0.5 μL ROX Reference Dye, 1 μL W chromosome-specific gene primer pair (5 μM each primer) (Table 1), 1 μL *PSMD4* gene primer pair (5 μM each primer), and 5 μL sterile water. In parallel, genomic DNA of adult females and males was included as positive control, whereas sterile water served as a negative control. PCR conditions were as follows: 95°C for 30 s, followed by 35 cycles of denaturation at 95°C for 5 s and annealing and extension at 60°C for 30 s. A dissociation step was performed to confirm the amplification specificity of each gene. All PCR amplifications were performed in biological triplicate. The PCR products were further examined by 2.5% agarose gel electrophoresis.

### Assessment of single- or mixed-sex schistosome infections in artificially infected snails by duplex qPCR

Each snail was immersed in water containing ~20 miracidia per well for 1 h in a 96-well plate. The snails were incubated at 26°C for 3 months and cercariae were released by one snail per well in a 24-well plate. The sex of six randomly selected cercariae from each snail was identified by the developed duplex qPCR method. To validate the accuracy of duplex qPCR, the sex of cercariae released from each snail was confirmed by morphologically distinguishable adult worms isolated from mice infected with cercariae from a single snail as described above.

### Field study

Fifty schistosome-infected snails were collected from a low-epidemic lake region in Hunan province, China. The sex of cercariae freshly released from each snail was identified by duplex qPCR as described above.

### Comparison of schistosomiasis mouse models based on sex-known and sex-unknown cercariae infection

For the sex-known infection group, the sex of freshly released cercariae was identified by duplex qPCR before infection. Each BALB/c mouse (6 weeks old, female, n = 8) was infected with 20 male and 20 female cercariae. For the sex-unknown infection group, each BALB/c mouse was infected with 40 ± 1 sex-unknown cercariae. At 6 weeks post-infection, adult worms were isolated from each mouse by hepatic-portal perfusion, and worm burdens were counted. Eggs were isolated from each liver by digestion with 4% KOH solution at 37°C for 24 h and eggs per gram of liver were counted [26]. For each group, two independent trials were performed.

### Statistical analysis

Statistical analysis was performed using GraphPad Prism 5.0 (GraphPad Software, CA, USA). Data were checked for normal distribution by the Shapiro-Wilk test. The data of two independent trials (total worm burden, sex ratio, worm pair burden and eggs per gram of liver) were compared by the unpaired Student’s *t*-test if the data followed a normal distribution. Non-normally distributed data were compared by the Mann-Whitney U test. *P* < 0.05 was considered as statistically significant.

## Results

### Identification of three W chromosome-specific genes

**Fig 1:**
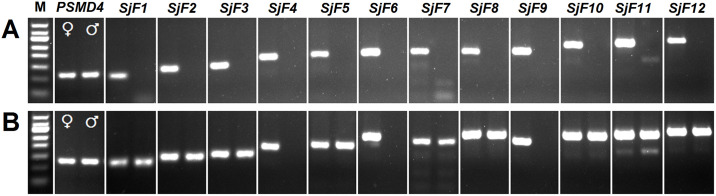
Identification of W chromosome-specific genes by end-point PCR. **(A)** End-point PCR using cDNA of adult females (♀, left lanes) and cDNA of adult males (♂, right lanes) as templates. **(B)** End-point PCR using genomic DNA of adult females (♀, left lanes) and genomic DNA of adult males (♂, right lanes) as templates. The expected sizes of PCR products are listed in Table 1. *PSMD4* was selected as a reference gene (129 bp). M represents a 50-bp DNA ladder marker.

The schistosome genome contains a pair of sex chromosomes. Females are the heterogametic sex with both Z and W chromosomes and males are homogametic with a ZZ pair. Therefore, genes localized exclusively on the W chromosome are ideal sex biomarkers, as they are expressed only in female worms. Based on microarray and bioinformatics analyses, 12 female-only expressed genes, encoding proteins with a variety of biological functions, were selected as candidate genes (Table 1). Several candidate genes or their homologous genes in *S*. *mansoni*, such as tyrosinase, female-specific protein 800, trematode eggshell synthesis domain-containing protein, and extracellular superoxide dismutase, have been shown to be expressed only in adult females [27–29]. The candidate gene *SjF12* annotated as tyrosinase (GenBank: AY815264.1) has been reported to play a critical role in eggshell formation [27, 30]. In this study, all 12 candidate genes were found to be expressed solely in females by reverse transcription PCR (Fig 1A). Three genes, including splicing factor U2AF 65 kDa subunit (*SjF4*; GenBank: FN317243.1), UV excision repair protein RAD23 (*SjF6*; GenBank: AY811322.1), and histone H3.3 (*SjF9*; GenBank: FN319455.1), were detected exclusively in the genomic DNA of adult females by end-point PCR (Fig 1B), suggesting that they were W chromosome-specific.

### Three W chromosome-specific genes are continually expressed in different developmental stages

**Fig 2:**
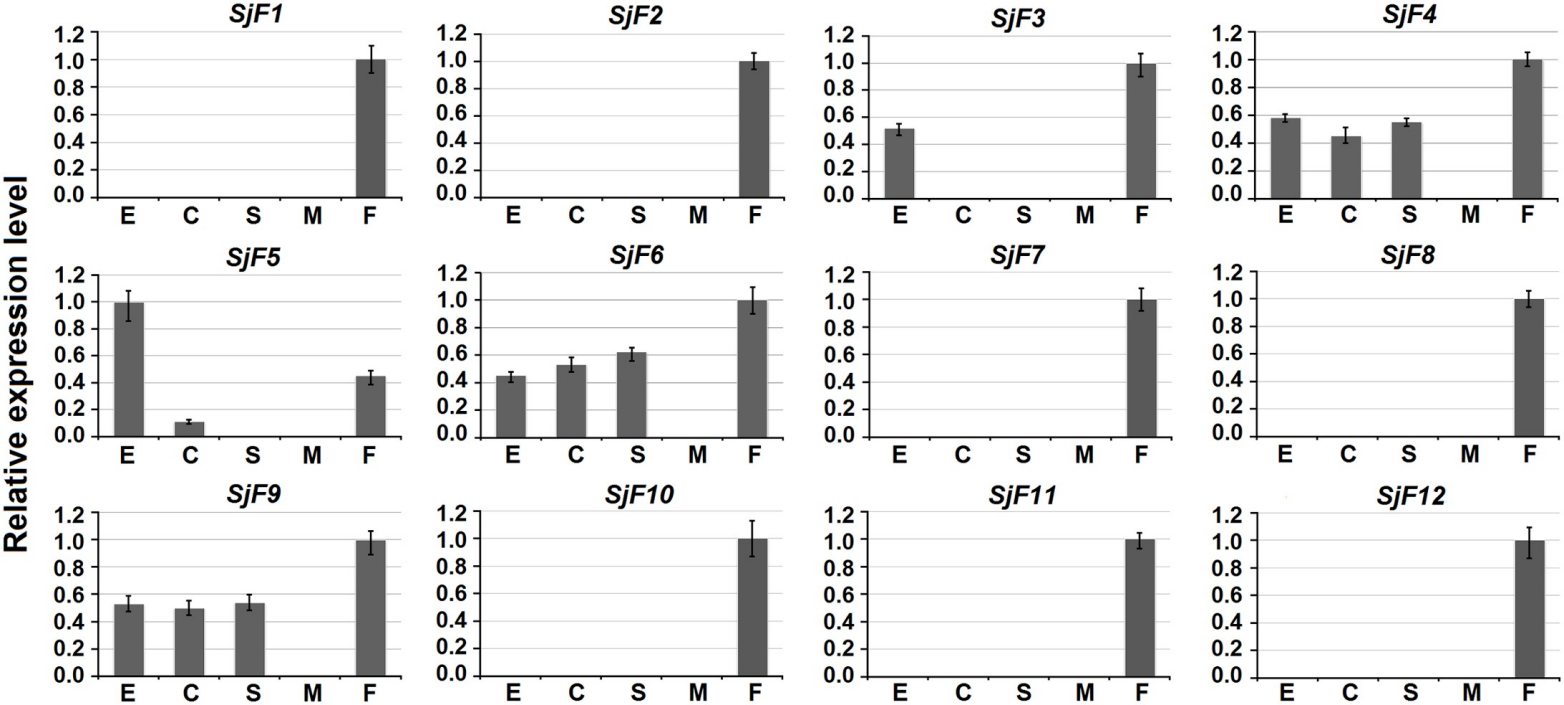
Expression patterns of 12 candidate genes in different developmental stages of *S*. *japonicum*. The expression levels of genes in four developmental stages (E, eggs; C, cercariae; S, hepatic schistosomula; M, adult male worms; F, adult female worms) were quantified by qPCR. The highest gene expression level in the four stages was set to 1. Error bars represent the standard deviation of three technical replicates.

Gene expression patterns of the 12 candidate genes in eggs, cercariae, hepatic schistosomula, adult male and female worms were analyzed by qPCR. Seven genes (*SjF1*, *2*, *7*, *8*, *10*, *11*, and *12*) were expressed exclusively in adult females. *SjF3* was expressed in both eggs and adult females. *SjF5* was expressed in eggs, cercariae, and adult females. Interestingly, the three W chromosome-specific genes (*SjF4*, *6*, and *9*) were expressed in all four developmental stages (Fig 2).

### Three W chromosome-specific genes are useful as novel sex biomarkers for different geographical populations of *S*. *japonicum*

**Fig 3:**
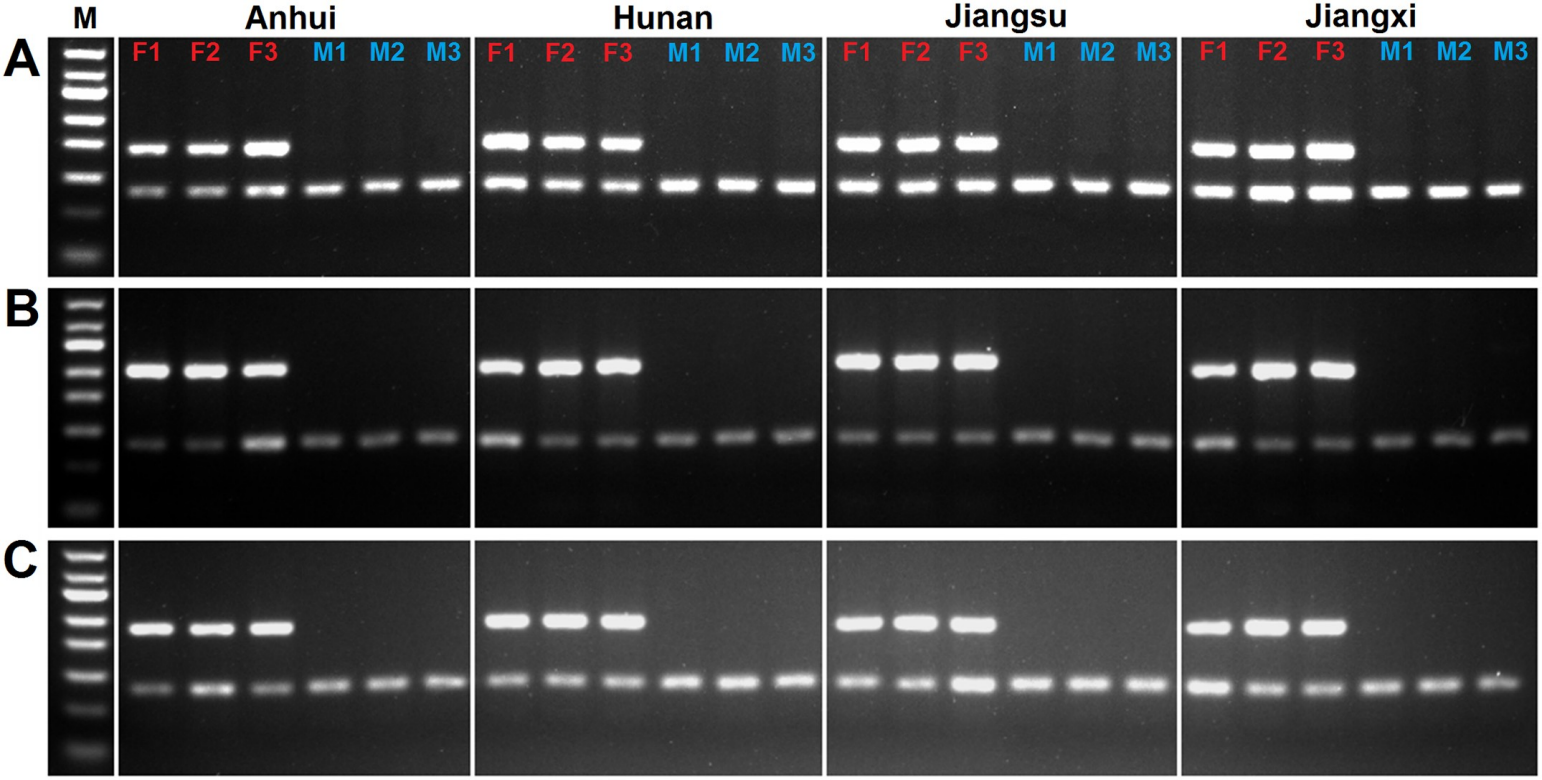
Three W chromosome-specific genes are useful as sex biomarkers of *S*. *japonicum* in different geographical populations. **(A)** Duplex PCR analysis of *SjF4* and *PSMD4*. **(B)** Duplex PCR analysis of *SjF6* and *PSMD4*. **(C)** Duplex PCR analysis of *SjF9* and *PSMD4*. The upper bands correspond to W chromosome-specific gene amplifications and the lower bands correspond to *PSMD4* amplifications. F1–3 represent biological triplicates of genomic DNA from adult females and M1–3 represent biological triplicates of genomic DNA from adult males. M represents a 50-bp DNA ladder marker.

A set of duplex PCRs was carried out using genomic DNA of adult worms from four different endemic regions in China. PCR products corresponding to the three W chromosome-specific genes were detected in the genomic DNA of all females from different geographic populations; whereas PCR products corresponding to *PSMD4* were detected in the genomic DNA of both males and females (Fig 3). This shows that the three primer pairs for W chromosome-specific genes can serve as reliable sex markers to identify the sex of cercariae among different *S*. *japonicum* populations.

### Establishment of an efficient duplex qPCR method for sexing cercariae

**Fig 4:**
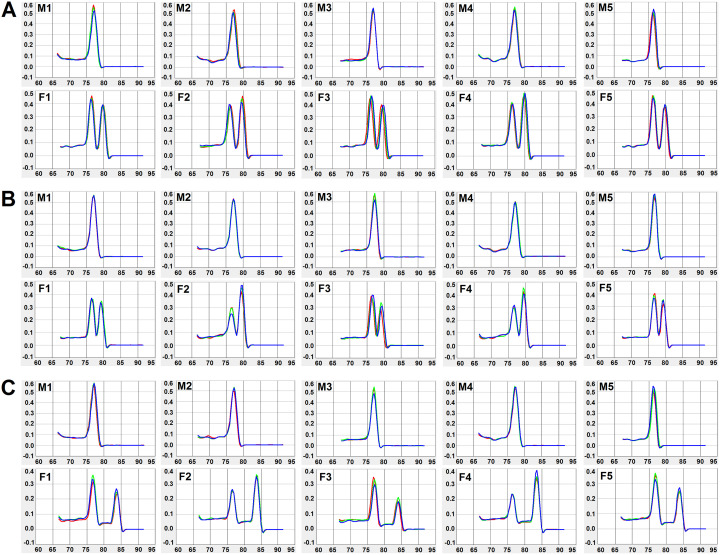
Duplex qPCR for sexing cercariae. Duplex qPCR melt curve analysis of W chromosome-specific gene markers *SjF4* (A), *SjF6* (B) and *SjF9* (C) using male and female cercariae. M1–5 represent male cercariae released from five snails; the single peak corresponds to the reference gene *PSMD4*. F1–5 represent female cercariae released from five snails; the two peaks correspond to *PSMD4* (first peak) and W chromosome-specific genes (second peak).

We used the above validated primer pairs to develop an efficient duplex qPCR method for sexing cercariae. Cercariae released from five male-schistosome-infected snails and five female-schistosome-infected snails were used as duplex qPCR models for sexing cercariae. Amplification curves of all cercaria samples were detected within 30 Ct values and no amplification curve was detected in the negative control (S3 Fig). All male cercaria samples exhibited a single peak and all female cercaria samples exhibited two peaks by melting curve analyses (Fig 4). Agarose gel electrophoresis analysis confirmed that all duplex qPCR products presented one (melt curves with single peak) or two (melt curves with two peaks) bands at the expected size (S4 Fig).

### The established duplex qPCR can be used to identify both single-sex and mixed-sex schistosome-infected snails

**Fig 5:**
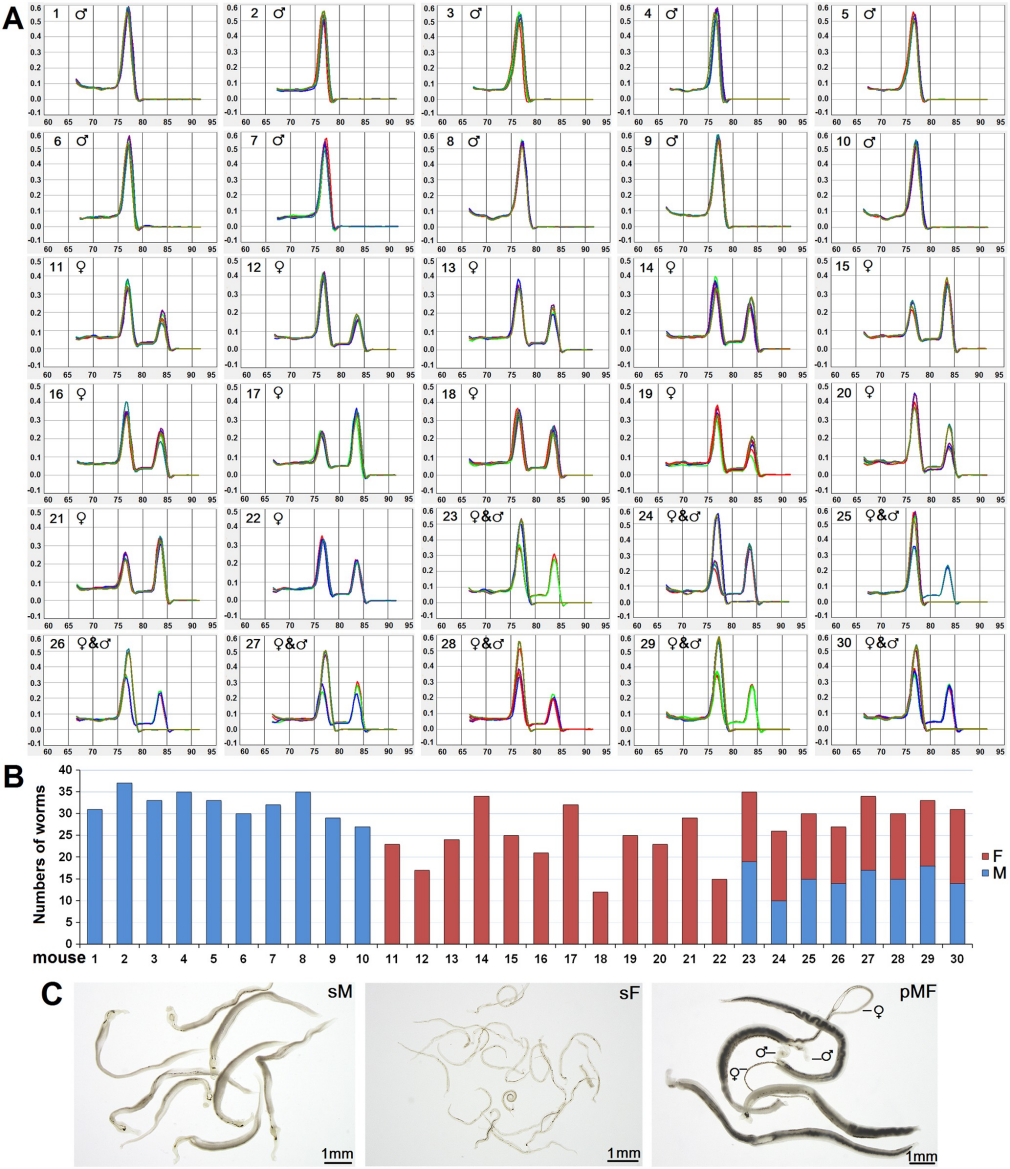
Single-sex and mixed-sex schistosome-infected snails are efficiently identified by duplex qPCR. **(A)** Duplex qPCR melting curve analysis of W chromosome-specific gene marker *SjF9* and *PSMD4* control for sexing cercariae released from 30 multi-schistosome infected snails. ♂ represents male cercariae, ♀ represents female cercariae, and ♀ & ♂ represents male and female cercariae. **(B)** Sex of cercariae released from the 30 snails was confirmed by adult worms isolated from corresponding infected mice. F represents adult females and M represents adult males. **(C)** Representative images of adult single-male worms (sM), adult single-female worms (sF), and adult male and female worm pairs (pFM). ♂ represents adult males, and ♀ represents adult females.

Usually, snails are exposed to dozens of miracidia to increase the success rate of infection. Here, we analyzed the sexes of cercariae released from multi-schistosome infected snails (n = 30) using the established duplex qPCR method. The results showed that 10 snails were infected with male schistosomes only, 12 were infected with female schistosomes only, and 8 were infected with both male and female schistosomes (Fig 5A). The accuracy of duplex qPCR for sexing cercariae was confirmed by recovering adult single-male, single-female, and mixed-sex worms from the corresponding infected mice (Fig 5B). Compared with single-male worms that undergo normal morphological development regardless of their pairing status, single-females remained immature and with a smaller body size than those paired with males (Fig 5C). These results demonstrate that the established method can be used to distinguish not only single-sex schistosome-infected snails but also mixed-sex schistosome-infected snails.

### Single-sex schistosome infections are prevalent among infected wild snails in a low-endemic lake region of China

Fifty schistosome-infected wild snails were collected from a low-endemic lake region and the sex of cercariae released from each snail was identified using the established duplex qPCR method. Twenty-three snails were infected with males, 25 snails were infected with females, and only 2 snails were infected with both male and female schistosomes (S5 Fig). The prevalence of single-sex schistosome infection in naturally infected snails was 96% (95% CI: 85.1–99.3%) and the sex ratio of schistosome infections was closed to 1 (23 males vs. 25 females).

### Standardized and reliable schistosomiasis mouse model using sex-known cercariae infection

**Fig 6:**
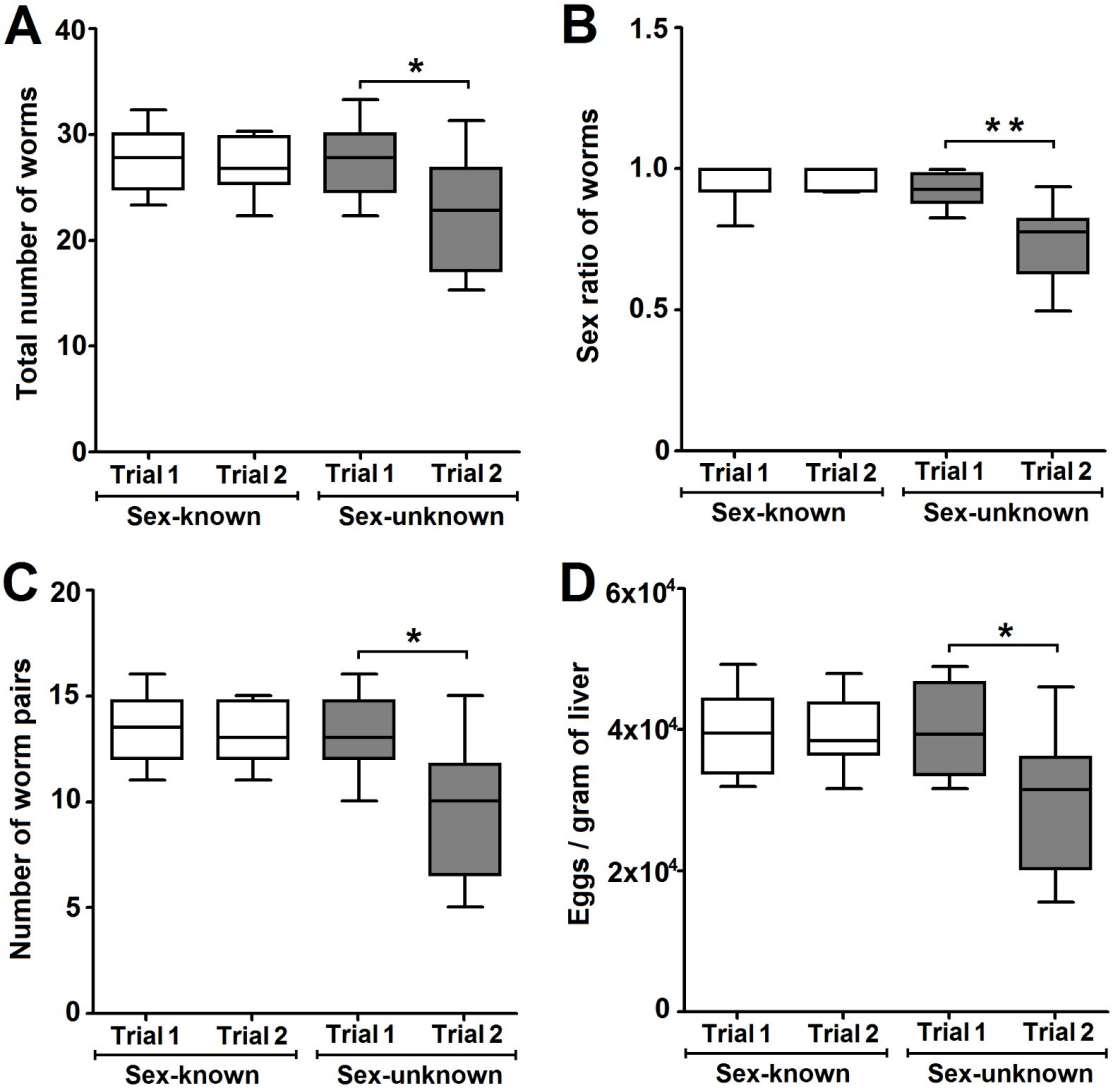
Reliable schistosomiasis mouse models using sex-known cercariae infection. Worm burdens **(A)**, sex ratio of worms **(B)**, worm pair burdens **(C)**, and egg burdens **(D)** of schistosomiasis mouse models from two independent trials. Horizontal lines represent the median values, and boxes and whiskers represent the inter-quartile and min-max ranges (n = 8), respectively. * represents *P* < 0.05, and ** represents *P* < 0.01.

The reliability of schistosomiasis mouse models based on sex-known or sex-unknown cercariae infection was compared. At 6 weeks post-infection, the total worm burden, sex ratio, worm pair burden, and eggs per gram liver from two independent trials for each model group were compared. No the parameters exhibited any significant difference between the two independent trials in sex-known cercariae-infected mouse models (*P* > 0.05, Fig 6), but significant differences were observed in sex-unknown cercariae-infected mouse models (*P* < 0.05, Fig 6). The sex ratio of adult worms was more balanced in trials of the sex-known infection group (0.96±0.03 and 0.97±0.01) compared with trials of the sex-unknown infection group (0.93±0.06 and 0.75±0.14) (Fig 6B). These results show that the schistosomiasis animal model based on sex-known cercariae infection was more controlled than the model based on sex-unknown cercariae infection.

## Discussion

*Schistosoma* species display a female heterogametic ZW/ZZ system, which is unique among more common hermaphroditic trematodes. Adult male and female worms display clear sexual dimorphism, whereas the sex of larvae (eggs, miracidia, sporocysts, cercariae, and schistosomula) is morphologically indistinguishable. Only a few molecular approaches for sexing *S*. *japonicum* larvae have been developed. Recently, Xu et al. [12] established a multiplex PCR method for sexing *S*. *japonicum* cercariae based on the female-specific DNA sequence reported by Zhao et al. [31]. Although the reported multiplex PCR could correctly identify male cercariae, the results for female cercariae were not accurate enough and mixed-sex cercariae could not be discriminated. Based on omics data mining, three *S*. *japonicum* W chromosome-specific genes were identified from 12 female-only expressed genes in this study. While unexpected, it is nevertheless reasonable that only three of the tested female-only expressed genes were W chromosome-specific. It is likely that either a female-specific transcription factor allows for transcription of these genes only in females, or a male-specific epigenetic imprinting on these genes represses their expression in males [32]. Based on the identified W chromosome-specific genes, an efficient duplex qPCR method for sexing *S*. *japonicum* cercariae was developed. The newly established duplex qPCR can identify the sex of cercariae within 2 h without DNA extraction, allowing further experiments to be performed on the same day as cercariae shedding. Alternatively, conventional end-point PCR based on the same primer pairs used for duplex qPCR can be employed to discriminate the sex of cercariae in a general laboratory. Moreover, duplex qPCR with a control primer pair for *PSMD4* as an autosome marker can overcome the shortcomings of traditional PCR, which cannot identify with certainty male cercariae in the absence of a PCR product and implies possible DNA extraction failure.

Schistosome genetic studies rely on mono-miracidial infection of snails to generate single-sex/single-genotype schistosome infections. The duplex qPCR will be a powerful tool for identifying sex of single-genotype cercariae for genetic researches, which can avoid the ethically questionable sacrifice of vertebrate hosts for sexing cercariae. Notably, this molecular method can also discriminate mixed-sex infection of snails. We used ~20 miracidia to infect one snail and the sexes of cercariae released from the infected snails were identified by duplex qPCR. The result showed that the proportions of male schistosome only, female schistosome only, and mixed-sex schistosome infections were nearly equal. Therefore, this method may be useful for generating single-sex/multiple-genotype infected snails for bulk segregant analysis.

Cercariae released from dozens of infected snails are routinely used to infect animals for schistosomiasis modeling in anti-schistosome drug or vaccine studies. The sex-ratio balance of worms is a major problem in schistosomiasis modeling because the sex of cercariae used for infection is generally unknown, yet it is critical for standardizing infection intensity and to control the progression of schistosomiasis [12]. Indeed, a significant sex-ratio imbalance of worms isolated from schistosomiasis mouse models has been found in previous studies [33–35], possibly interfering with the evaluation of drug or vaccine effectiveness. In this study, we compared the reliability of schistosomiasis mouse models based on sex-known and sex-unknown cercariae infections. The result showed that the total worm burden, sex ratio of worms, worm pair burden, and eggs per gram liver between independent trials were more controlled between sex-known infected mice than between sex-unknown infected mice. The numbers of adult worms recovered from sex-unknown cercariae-infected mice can fluctuate widely, particularly if the mice are infected with female-biased cercariae. Compared with paired adult worms, tiny virgin females are more difficult to retrieve by perfusion because they may be easily stuck in the host’s liver or mesenteric veins and are thus easily missed.

Molecular methods for sexing cercariae will not only improve the reliability of schistosomiasis animal models for anti-schistosome drug or vaccine studies, but also revolutionize clinical research on this devastating disease. Traditionally, the protective efficacy of vaccine candidates should be confirmed by long-term and/or large-scale field trials in endemic areas after clinical phase 1 testing for safety. To reduce the risk of downstream efficacy failure, healthy volunteers are intentionally exposed to pathogens to test infectious disease vaccines [36]. Such controlled human infection trials have provided valuable efficacy forecasts to guide further clinical development. The main adverse effect of schistosomiasis arises from immunopathological reactions against eggs rather than adult worms trapped in the tissues or organs, which lead to serious intestinal, hepato-splenic or urogenital disease [2, 37]. Therefore, it is impracticable to test vaccine protection against schistosomes based on mixed-sex cercariae infection in clinics due to egg-associated immunopathological damage. To ensure the safety of participants in clinical trials, a molecular approach helped to establish a controlled human schistosomiasis model for clinical vaccine research based on male *S*. *mansoni* cercariae infection [38, 39]. The availability of controlled human infection model for schistosome infection may revolutionize the development of schistosomiasis vaccines in the future. In addition, a growing number of studies have been performed to investigate the influence of unisexual schistosome infection on the host’s immune system and intestinal microbiota, which provide a promising prospect for new immune modulatory therapies [40–42].

After decades of comprehensive prevention and control, great success has been achieved against schistosomiasis japonica in China. The prevalence and intensity of schistosome infections in snails and humans have been reduced to extremely low levels, and the national schistosomiasis control program has shifted its goal from transmission control to transmission interruption and elimination since 2015 [43]. Based on final host mouse infection, Shi et al. found that most infected snails in endemic hilly areas of China harbored clonal single-sex schistosomes [44]. Using the established duplex qPCR, we found that single-sex schistosome infections were predominant among infected snails in endemic lake areas of China. However, the sex ratio of cercariae may be affected by total numbers of cercariae released from field snails since only 6 cercariae from each snail were randomly selected to test for the sex. Quantifying the proportion of autosomal and allosomal markers of total cercariae released from snails may give a more accurate view of the sex ratio. Moreover, single-sex schistosome infections have been reported in sentinel mice used to detect schistosomiasis transmission sites along the Yangtze River in China [45]. An eight-year ecological study of natural populations of *S*. *mansoni* found that a fifth of schitosome-infected wild rats harbored single-sex schistosome infections in Guadeloupe [46]. These studies suggest that single-sex schistosome exposures and subsequent infections are becoming more common among definitive hosts in low-epidemic areas, which can strongly influence schistosomiasis epidemiology and evolution of *Schistosoma* species. As a refuge for schistosomes, single-sex infected definitive hosts cannot be identified by traditional egg-based parasitological detection methods. Initial single-sex schistosomes can pair and viably reproduce with subsequent invading specimens of the opposite sex, thus continuing disease transmission [47]. Due to increasing human migration and economic globalization, feasible hybridization between different *Schistosoma* species, particularly between livestock and human *Schistosoma* species, will represent an emerging and profound public health threat towards global schistosomiasis elimination [48, 49]. Indeed, indisputable evidence for hybridization among *S*. *haematobium*, *S*. *mansoni*, *S*. *bovis*, *S*. *currassoni*, and *S*. *intercalatum* has been provided by numerous studies [50–55]. The outbreak of urogenital schistosomiasis caused by *S*. *haematobium*-*bovis* hybrids in France demonstrates the public health impact of such introgressive hybridization [52]. Growing numbers of schistosomiasis haematodia and mansoni imported from Africa have been reported in China because of the rapid growth of China-supported projects and labor services in Africa [56, 57]. More attention should be paid in the future to the surveillance of feasible interspecific hybrids between *S*. *japonicum* and *S*. *mansoni* or *S*. *haematobium*.

In summary, we developed a highly efficient duplex qPCR approach for discriminating the sex of *S*. *japonicum* cercariae. The proposed method is valuable for establishing unisexual or mixed-sex schistosome-infected mouse models for anti-schistosomal drug, vaccine, and host-parasite interplay studies. Moreover, the newly developed duplex qPCR method can greatly improve the identification efficiency of unisexual or mixed-sex schistosome-infected snail models. Finally, this molecular method can be extensively applied in molecular epidemiological field studies of unisexual or mixed-sex schistosome-infected snails.

## Supporting information

S1 Fig Agarose gel electrophoresis analysis of PCR products from the negative control. PCR was performed using DNase-treated RNA of male (♂, right lanes) and female (♀, left lanes) worms as templates. No PCR products of *PSMD4* and selected candidate genes (Table 1) were detected. M represents a 50-bp DNA ladder marker.
(PNG)

S2 Fig Melting curves of 12 candidate genes validated by qPCR. (PNG)

S3 Fig Duplex qPCR amplification curves of all cercaria samples can be detected within 30 Ct values. **(A)** Duplex qPCR amplification curves of *SjF4* and *PSMD4*. **(B)** Duplex qPCR amplification curves of *SjF6* and *PSMD4*. **(C)** Duplex qPCR amplification curves of *SjF9* and *PSMD4*. Cer represents duplex qPCR amplification curves of cercariae and Neg represents duplex qPCR amplification curves of negative controls.
(PNG)

S4 Fig Agarose gel electrophoresis analysis of duplex qPCR products. **(A)** Duplex qPCR products of *SjF4* and *PSMD4*. **(B)** Duplex qPCR products of *SjF6* and *PSMD4*. **(C)** Duplex qPCR products of *SjF9* and *PSMD4*. The upper bands correspond to W chromosome-specific gene amplifications and the lower bands correspond to *PSMD4* amplifications. F1–5 represent five biological replicates of female cercariae and M1–5 represent five biological replicates of male cercariae. M represents a 50-bp DNA ladder marker.
(PNG)

S5 Fig Duplex qPCR for sexing cercariae released from infected snails in a field study. Duplex qPCR melt curve analysis of W chromosome-specific gene marker *SjF9* and *PSMD4* control for sexing cercariae released from 50 naturally infected snails. ♂ represents male cercariae, ♀ represents female cercariae, and ♀ & ♂ represents male and female cercariae.
(PNG)
