# Supplementary figures and images for: Duplex real-time PCR for sexing Schistosoma japonicum cercariae based on W chromosome-specific genes and its applications
Source: PLoS Negl Trop Dis. 2020 Aug 21;14(8):e0008609. doi: 10.1371/journal.pntd.0008609 (PMC7467314; doi:10.1371/journal.pntd.0008609)

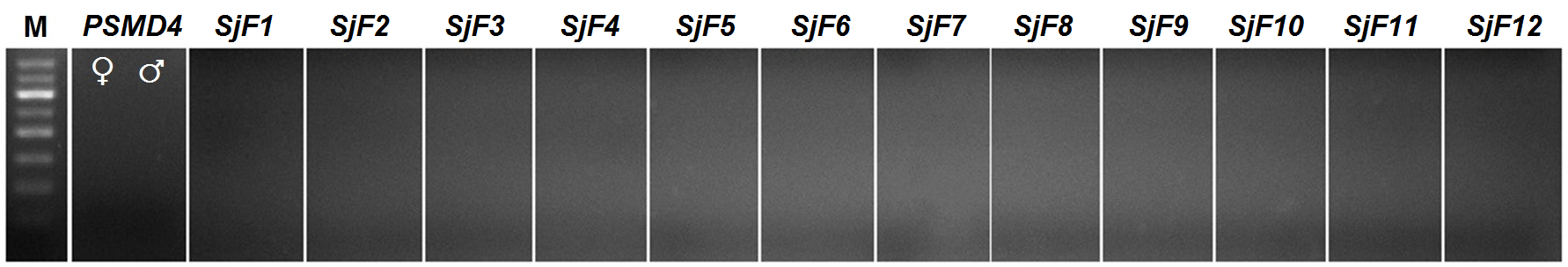

Supplement: S1 Fig — PCR was performed using DNase-treated RNA of male (♂, right lanes) and female (♀, left lanes) worms as templates. No PCR products of PSMD4 and selected candidate genes (Table 1) were detected. M represents a 50-bp DNA ladder marker. (PNG) [file pntd.0008609.s001.png]

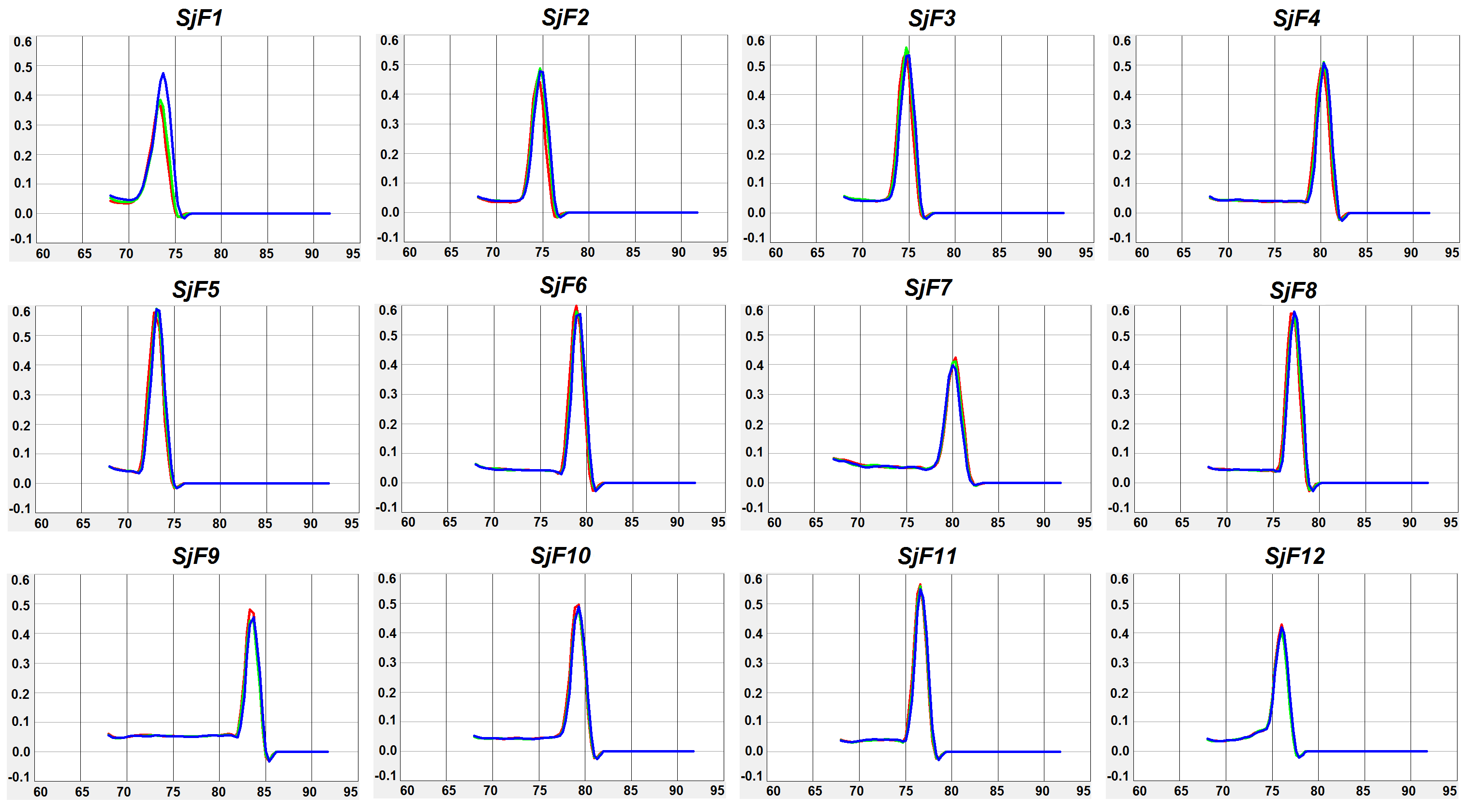

Supplement: S2 Fig — (PNG) [file pntd.0008609.s002.png]

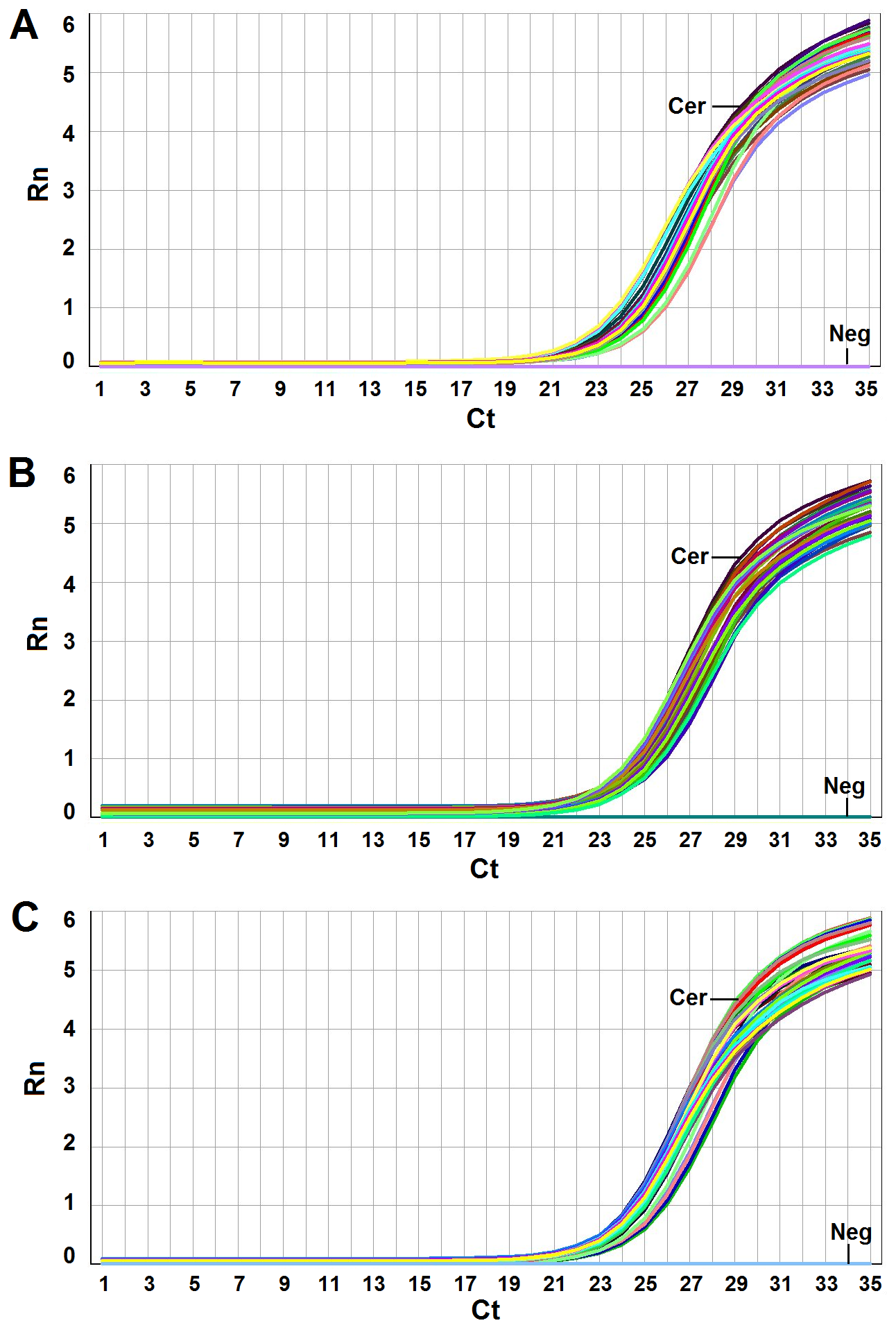

Supplement: S3 Fig — (A) Duplex qPCR amplification curves of SjF4 and PSMD4. (B) Duplex qPCR amplification curves of SjF6 and PSMD4. (C) Duplex qPCR amplification curves of SjF9 and PSMD4. Cer represents duplex qPCR amplification curves of cercariae and Neg represents duplex qPCR amplification curves of negative controls. (PNG) [file pntd.0008609.s003.png]

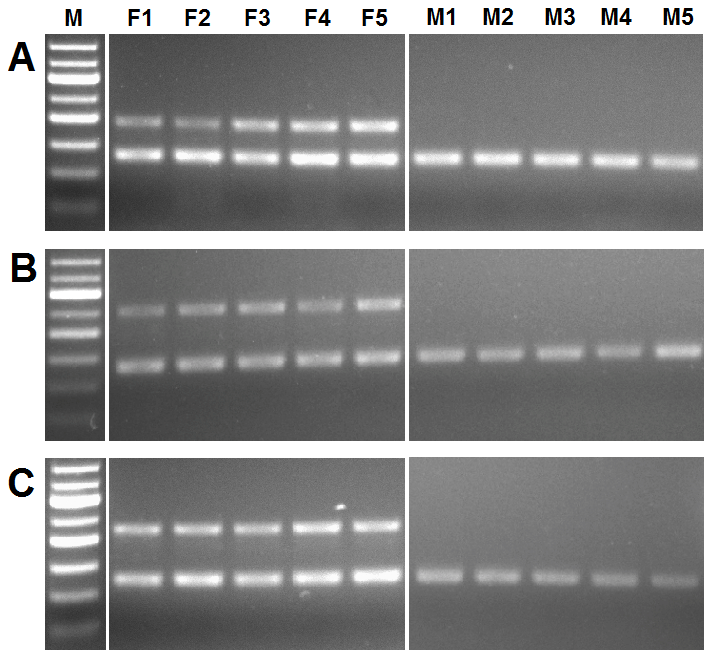

Supplement: S4 Fig — (A) Duplex qPCR products of SjF4 and PSMD4. (B) Duplex qPCR products of SjF6 and PSMD4. (C) Duplex qPCR products of SjF9 and PSMD4. The upper bands correspond to W chromosome-specific gene amplifications and the lower bands correspond to PSMD4 amplifications. F1–5 represent five biological replicates of female cercariae and M1–5 represent five biological replicates of male cercariae. M represents a 50-bp DNA ladder marker. (PNG) [file pntd.0008609.s004.png]

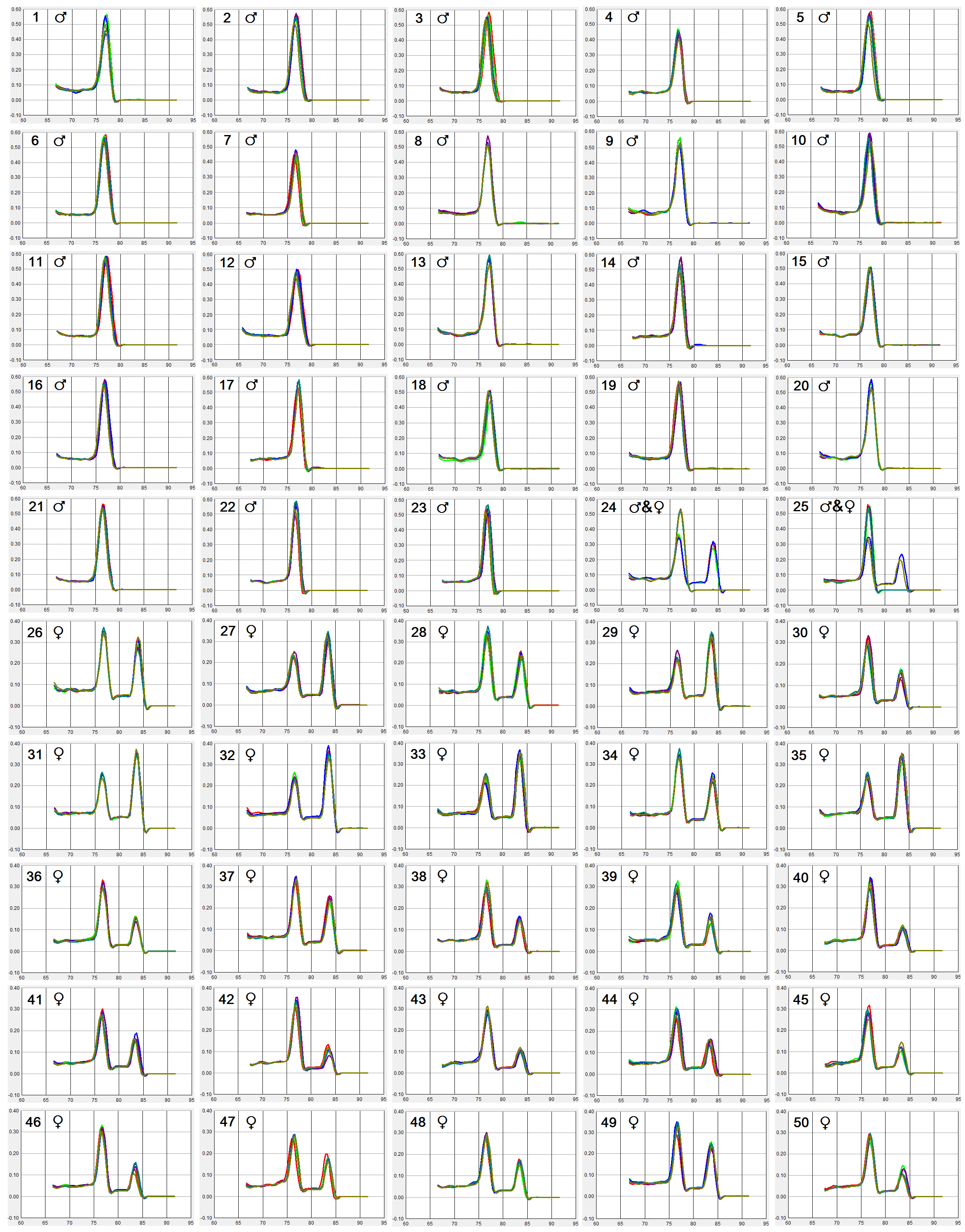

Supplement: S5 Fig — Duplex qPCR melt curve analysis of W chromosome-specific gene marker SjF9 and PSMD4 control for sexing cercariae released from 50 naturally infected snails. ♂ represents male cercariae, ♀ represents female cercariae, and ♀ & ♂ represents male and female cercariae. (PNG) [file pntd.0008609.s005.png]
